# Changes in Local Government Priorities and Stakeholder Satisfaction Following a Healthy Drinks Capacity‐Building Intervention in Sports and Recreation Facilities in Victoria, Australia

**DOI:** 10.1002/hpja.70024

**Published:** 2025-02-24

**Authors:** Miranda R. Blake, Devorah Riesenberg, Tara Boelsen‐Robinson, Adrian J. Cameron, Anna Peeters

**Affiliations:** ^1^ Institute for Health Transformation, Global Centre for Preventive Health and Nutrition, School of Health and Social Development, Faculty of Health Deakin University Geelong Australia; ^2^ Institute for Health Transformation Deakin University Geelong Australia

**Keywords:** Australia, local government, nutrition policy, sports

## Abstract

**Issue Addressed:**

Local government (LG) facilities such as libraries, clubs and sport and recreation centres generally offer and promote nutritionally poor foods that may contribute to excess energy intake. It is unclear what effect exposure to a healthy food retail initiative may have on customer and staff support for such changes. This study investigated the association of a healthy drink intervention in LG‐owned sporting facilities on changes in (i) intervention LG attitudes and facility healthy food and drink provision, compared to control LGs; and (ii) intervention LG customer and staff support for the intervention.

**Methods:**

Eight Victorian LGs implemented a two‐year intervention to limit sugary drink availability in LG‐owned sporting facilities. At baseline (2018) and follow‐up (2020), attitudinal surveys were sent to intervention LG staff (baseline: *n* = 162; follow‐up: *n* = 183) and customers (baseline: *n* = 1079; follow‐up: *n* = 1188), and policy surveys were sent to all state LGs. Baseline and follow‐up responses, and policy survey responses between 8 intervention and 24 control LGs were compared using logistic regression.

**Results:**

Intervention LGs' baseline priorities for promoting healthy eating were higher than control LGs, with no change in priorities at follow‐up. Intervention LG staff and customers' belief that sporting facilities should promote healthy eating remained high between baseline (customers 79%; staff 76%) and follow‐up (customers 75%; staff: 73%).

**Conclusions:**

Facility staff and customers support interventions to align sporting facilities' healthy lifestyle message with healthier food environments.

**So What?:**

LG prioritisation of healthy eating appears to be a prerequisite for LG healthy drink policies.

## 1 Introduction

An all‐of‐government approach is needed to improve the health and wellbeing of communities. Community food retail settings, such as those located in libraries, clubs and sport and recreation facilities, present a key opportunity to support health by providing customers with healthy food and drink options. Local government (LG) is uniquely placed to provide infrastructure to support physical activity (e.g., parks, walking paths and sporting facilities) and adopt regulation to ensure healthy food and drink provisions (e.g., requiring healthy food and drink options be available at LG‐owned facilities) [1].

There is increasing recognition of the role LGs play in community health, including through policy relating to retail outlets in LG‐owned facilities [2, 3, 4]. LGs worldwide are increasingly implementing regulations and interventions to promote health [5, 6, 7, 8, 9]. A 2020 survey of Australian LGs found 22% of LGs reported that the priority they gave to promoting healthy food had increased from the previous year [10]. Ten percent of LGs had a formal policy on healthy food and drink provision at sport and recreation facilities, while 32% had made healthy changes without a formal policy [10]. While it is generally assumed that policy change comes before practice change, there is currently no evidence on how LG policy priorities *change after* the implementation of a healthy food intervention.

Interventions where values between those involved are aligned and where trust between partners is present have an increased probability of success and being sustained over time [11]. It is often the retail staff, including managers and chefs, who are responsible for implementing and supporting healthy food retail changes. Change is dependent on their beliefs regarding the need for and efficacy of change [12] and their support for change [13, 14, 15]. A 2020 study evaluating pricing interventions to promote healthy food and drink choices, such as meal deals, in four Victorian aquatic and recreation facilities found that managers' beliefs and attitudes towards the (often opposing) goals of health promotion versus profit were associated with acceptance of the intervention [15]. To date, we are aware of only one study that has reported on retail staff commitment to change over time in a healthy food retail intervention [4]. That study of 17 interviews with six LG stakeholders conducted over the course of an LG intervention found an increase over time in optimism regarding the intervention. The study highlighted the value of examining pre‐ and post‐intervention opinions, as these are likely to change after intervention exposure and affect maintenance of the intervention and engagement in future healthy changes.

A key reason for retail manager reluctance to implement healthy changes is the belief that customers do not want healthier options [11, 13, 16]. Managers may fear that if they do not provide popular unhealthy options, customers will go elsewhere and thus reduce their sales. Low‐ to moderate‐quality customer surveys have found consistently high support for healthy changes such as limiting the amount of sugary drinks available following healthy food retail interventions in sporting facilities [17, 18, 19]. It is unclear whether these results reflect an increased likelihood of implementing healthy changes in areas with high pre‐existing customer acceptance, or an increase or maintenance of support following exposure to an intervention. No studies have yet reported on the pre‐post changes in customer opinions in response to exposure to a healthy food retail initiative in sporting settings.

The aim of the current study was to evaluate the impact of the ‘Water in Sport’ (WIS) LG healthy drinks intervention in sports and recreation facilities in Victoria, Australia on change in (i) LG policies, attitudes, and practices relating to obesity prevention and provision of healthy food and drink in sporting facilities, compared to comparison control LGs in which no intervention took place; and (ii) customer and staff support for the intervention within intervention LGs.

## 2 Methods

### 2.1 Setting

The WIS initiative ran from January 2018 to March 2020. It provided human resourcing and training to LGs to make water the drink of choice in local sport and recreation facilities. Eight LGs in Victoria, Australia, were funded by the Victorian Health Promotion Foundation (‘VicHealth’) to each employ a project officer for a 2‐year period. Project officers were employed at all LGs between July 2018 and December 2019, in order to increase council capacity to make changes, with staggered start and finish dates ranging from March 2018 to June 2020. LGs self‐selected to apply to participate in the initiative and were funded at the discretion of VicHealth. Project officers were responsible for facilitating council‐level healthy drinks policy changes and the implementation of one of two ‘nudges’ in LG‐owned and/or ‐managed sporting facilities in each LG (total 56 participating facilities; range 3 to 10 per LG). LGs nominated as many facilities expressing interest to participate as they wished. Nudges were intended to increase the provision and purchasing of healthier drink options and consisted of either (i) limiting sugar‐sweetened drinks to less than 20% of fridge display space, or (ii) removing sugar‐sweetened drinks from fridge display. Drink healthiness classifications and nudge targets were aligned with the voluntary state government ‘Healthy Choices: policy guidelines for sport and recreation centres’ [20]. Project officers negotiated with facility managers to decide which ‘nudge’ would be implemented within each facility kiosk, café, canteen or vending machine. Examples of tailored project officer support included identifying alternative suppliers, developing healthy fridge planogram (layout designs) and staff training. Further details on the project design can be found in an evaluation of the implementation of the initiative and its impact on the healthiness of drinks sold and facility revenue [21].

### 2.2 Study Design

This convergent multi‐method non‐randomised study included pre‐post surveys of three stakeholder groups (LG employees, facility staff and facility customers) to understand how policy, and staff and customer knowledge, attitudes and behaviours changed after exposure to the WIS initiative. The data sources are presented separately in the results below, and then the complementary findings are brought together in the discussion to develop a comprehensive understanding of the impact of the intervention from different stakeholder perspectives. The LG policy survey is presented first to provide the overall policy context and the demographic characteristics of WIS intervention and control LGs. Table 1 summarises the aims, methods, timing and sample recruitment for each survey.

**TABLE 1:** Summary of surveys used in the evaluation of the Water in Sport initiative, in local government‐owned sporting facilities, Victoria, Australia, 2018–2020.

Data source	Aim	Method (brief)	Timepoints measured	Participants and recruitment
Local government surveys	To compare change in policies, attitudes, and practices of Water in Sport and other local governments in Victoria relating to obesity prevention and the provision of healthy food in their sport and recreation facilities	Repeat cross‐sectional survey assessing local governments' healthy food and drink provision policies relating to sport and recreation facilities and the priority given by local governments to obesity prevention	Baseline: July 2018 Follow‐up: July 2020	All local governments in the Australian state of Victoria (*n* = 79) were invited (Baseline: *n* = 49; Follow‐up: *n* = 37)
Customer surveys	To assess facility customer awareness, and satisfaction with healthy drink changes and perceptions of the need for change	Repeat cross‐sectional customer exit and online surveys assessing sociodemographic characteristics, purchasing patterns, awareness and attitudes towards the intervention and need for the intervention	Baseline: June‐September 2018 Follow‐up: June‐September 2019	Convenience sample of customers attending or on a mailing list for facilities within Water in Sport intervention LGs only (Baseline: *n* = 1079; Follow‐up: *n* = 1188)
Staff surveys	To assess staff awareness, and satisfaction with healthy drink changes and perceptions of the need for change	Repeat cross‐sectional online surveys on staff purchasing patterns and attitudes towards intervention and need for the intervention	Baseline: June‐September 2018 Follow‐up: June‐September 2019	Convenience sample of key stakeholders involved in policy development and implementation of the Water in Sport intervention within intervention LGs only (Baseline: *n* = 162; Follow‐up: *n* = 183)

### 2.3 Local Government Survey

An online cross‐sectional survey was emailed to the generic email addresses of all 79 Victorian LGs in July 2018 and again in July 2020 addressed ‘ATTN Health and Wellbeing Manager’. Questions were informed partly by a previously developed policy implementation and adoption survey designed for sport and recreation facilities in Canada [14]. Survey questions explored (i) the types of LG owned and/or managed facilities that sold food or drink, and any improvement to the healthiness of food and drink provision to date; (ii) the priority given by LGs to obesity prevention and the removal of sugary drinks from facilities (on an ordinal scale between 0 and 10) and (iii) barriers and enablers to change (results not reported). For the follow‐up survey, minor changes were made to the baseline survey [9] based on stakeholder feedback. See Supporting Information: Appendix 1 for full baseline and follow‐up surveys.

The analysis for this study was restricted to the intervention and control LGs (those not involved in the WIS intervention) who completed surveys at both baseline and follow‐up to facilitate longitudinal comparison of changes over time. Analysis relating to the baseline data collected on government action and policy in all Victorian LGs surveyed has been previously published [9, 10], including a comparison with expressed support for change versus current LG policy [9]. LG community characteristics were compared for responding intervention and control LGs and non‐responding Victorian LGs for socioeconomic position (measured using deciles of the Socio‐Economic Indexes for Areas (SEIFA) [22] with 1 indicating the highest level of disadvantage and 10 indicating the most advantage); population size [23]; remoteness (measured using the Australian Bureau of Statistics classifications of major cities, inner regional, outer regional, and remote/very remote) [24]. Categorical data were compared between intervention and control LGs at each timepoint using Wilcoxon rank‐sum tests (for ordinal or non‐parametric continuous data) or Pearson chi‐squared tests. Changes in the priority given to healthy eating between survey time points and between intervention and control LGs were assessed using multivariate linear regression with clustering at the LG level.

### 2.4 Customer and Staff Surveys

Repeat cross‐sectional customer and staff surveys were conducted at baseline (June–September 2018) and follow‐up (June–September 2019) in the eight intervention LGs. An individual survey link was sent at follow‐up to respondents who provided their email at baseline.

Customer surveys were completed online or in‐person by customers frequenting sporting facilities in intervention LGs. Recruitment was via both customer email lists and paper copies of surveys left at food outlets. The survey investigated the perceived need for healthy drink options, awareness of healthy drinks changes and customer knowledge related to the healthiness of sugary drinks, as well as customer demographics (see Supporting Information: Appendix 2 for full survey).

Staff surveys were completed by individuals involved in policy development related to the nudges, the implementation of nudges and those that the policy would influence (i.e., facility staff). Staff were recruited by WIS project officers via staff email lists. The survey investigated the perceived need for healthy drink options, the awareness of healthy drinks policy implementation, staff opinion on organisational intent to improve the overall healthiness of offerings, and whether the staff member was personally involved in the healthy retail changes (see Supporting Information: Appendix 3 for full survey).

Customer and staff survey responses were included for analysis if all non‐demographic questions had been completed. Characteristics of participants at baseline and follow‐up were compared using chi‐squared tests. Logistic regression or ordered logistic regression was used to compare responses at the two timepoints, as appropriate. Customer surveys were adjusted for clustering at the LG and facility level. Survey sensitivity analyses included (i) restricting analysis to only those facilities with no nudges at baseline; (ii) where the nudge of limiting sugary drinks to < 20% of drinks display was implemented at follow‐up (34/54 facilities) and (iii) removing responses where the facility was unknown (i.e., staff and customers did not select what facility they worked at/most frequented on the paper‐based survey). Only significant differences between baseline and follow‐up results are reported in the text (*p* < 0.05).

All statistical analyses for LG, customer and staff surveys were completed in Stata 15 (StataCorp, TX USA).

### 2.5 Ethical Approval

This study was conducted according to the guidelines laid down in the Declaration of Helsinki, and all procedures involving research study participants were approved by the Deakin University Faculty of Health Human Ethics Advisory Group (reference HEAG‐H 35_2018). Implied informed consent was obtained from all subjects through the completion of the surveys.

## 3 Results

### 3.1 Local Government Survey

#### 3.1.1 Participating Local Government Demographic Characteristics

A total of 41 of 79 Victorian LGs completed the survey at baseline (52% response rate), and 37 of 79 at follow‐up (47% response rate). Thirty‐two LGs (8 intervention LGs and 24 control LGs) completed surveys at both baseline and follow‐up. LGs who responded to the survey were reflective of overall Victorian LG characteristics regarding SEIFA and geographic location (as assessed at baseline). The average population size was greater in both intervention and control LGs compared to non‐participating LGs (*p* < 0.05). No statistical differences were found in intervention and control LGs in relation to level of socioeconomic disadvantage, population size and rurality (Table 2).

**TABLE 2:** Demographic characteristics of Water in Sport intervention and control local governments (*n =* 32) and all Victorian local governments (*n* = 79).

Characteristic	All Victorian LGs (*n* = 79)	Participating intervention LGs (*n* = 8)^a^	Participating control LGs (*n* = 24)^a^
Median [interquartile range]			
SEIFA decile	5 [3, 8]	5 [2.5, 6]	5.5 [3, 8.5]
Population size	45 040 [15 952; 134 143]^b^	130 368 [56 907; 162 178.5]	70 955.5 [18 313.5; 167 918.5]
*n* (%)			
Location			
Major cites	33 (42)	4 (50)	12 (50)
Inner‐regional areas	33 (42)	3 (38)	10 (42)
Outer‐regional areas	13 (16)	1 (13)	2 (8)

Abbreviations: LG, local government; SEIFA, Socio‐Economic Indexes for Areas.

^**a**^ Includes LGs who completed surveys at both time points only.

^b^ Difference between participating intervention and control LGs, and remaining Victorian LGs *p* = 0.0469.

#### 3.1.2 Local Government Priorities

LG priorities were higher in intervention than control LGs at baseline regarding: ‘promoting healthy eating/drinking’ (difference +2.1 [95% CI: +0.7, +3.5] points on 11‐point scale from 0 to 10); ‘increasing the availability of healthy food/drink in LG owned sport and recreation facilities’ (difference +3.6 [+1.9, +5.3]) and ‘reducing the availability of sugary drinks for sale in LG owned sport and recreation facilities’ (difference +3.2 [+1.3, +5.1]) (all *p* < 0.04; see Supporting Information: Appendix 4, Table S1). There was no difference between intervention and control LGs in the priority to ‘reduce the prevalence of obesity’ at baseline. At follow‐up, LG priorities related to ‘improving public health and wellbeing’ were high in both intervention (8.4 [7.3, 9.5]) and control LGs (8.7 [8.0, 9.3]), with no significant difference between groups (this variable was not collected at baseline). There was no significant intervention effect between intervention and control LGs between baseline and follow‐up in any of the four priority areas (See Supporting Information: Appendix 4, Table S1).

#### 3.1.3 Healthy Food Retail Action and Policy Adoption

All eight intervention LGs had made healthy food retail changes to either drinks only (25% intervention LGs) or both food and drink (75% intervention LGs) at the baseline survey. This presents a ceiling effect to intervention LG action that was not present for control LGs at baseline (33% LGs no changes made; 21% changes to drinks only; 46% changes to both food and drink). No differences were found between intervention and control LGs in actions taken to improve the healthiness of food and/or drink provision in sport and recreation facilities at either time point (Table 3). At follow‐up, four intervention LGs (50%) and four control LGs (17%) had official policies relating to any aspect of healthy drinks provision. A further 13 control LGs had taken action to discourage sugary drinks in the absence of official written policies, or additional actions beyond written policies. These actions included increasing the availability of water both free and for purchase (*n* = 10 LGs), no advertising of sugary drinks (*n* = 10), sugary drinks hidden from display (*n* = 4), and no sugary drinks allowed to be sold (*n* = 4).

**TABLE 3:** Comparison of healthy food retail action in Water in Sport intervention (*n* = 8) and control (*n* = 24) local governments^a^.

Action taken	Intervention local governments (*n* = 8)		Control local governments (*n* = 24)	
	*n* (%)			
	Baseline	Follow‐up	Baseline	Follow‐up
No change made	0 (0)	0 (0)	8 (33)	9 (38)
Change to drinks only	2 (25)	1 (12)	5 (21)	1 (4)
Change to food only	0 (0)	0 (0)	0 (0)	0 (0)
Change to both food and drink	6 (75)	7 (88)	11 (46)	14 (58)

^a^ No statistically significant differences were found between intervention and control councils at either baseline or follow‐up.

### 3.2 Customer Survey

#### 3.2.1 Customer Demographic Characteristics

The customer survey was completed by 1079 customers at baseline and 1188 customers at follow‐up, with 90 customers completing surveys at both timepoints (respondent characteristics presented in Supporting Information: Appendix 4, Table S2). Three hundred and fifty‐seven surveys were excluded from analysis as their location was unknown or their facility was not actively involved in the WIS intervention. At both time points, most customers who completed the survey were female, aged between 25 and 44 years, and had completed at least high school level of education. Most customers who completed the survey purchased food or drink at the retail outlet in the sport and recreation facility 1–4 times per week (baseline 46.9%; follow‐up: 47.1%) or never (baseline 35.6%; follow‐up: 37.4%).

#### 3.2.2 Customer Support for and Awareness of Intervention

Customer support was high at baseline for the statements ‘sport and recreation facilities have a responsibility to promote healthy eating’ (76.9%) and ‘removing sugary drinks from sport and recreation facility would lead to reduced consumption in the community’ (54.2%), with no difference in these percentages between baseline and follow‐up. At follow‐up, a slightly higher proportion of customers had noticed a change in the past 6 months in the variety (baseline: 4.0%; follow‐up: 6.0%) and availability (3.7%; 7.8%) of sugary drinks, as well as changes to the availability (1.9%; 4.1%) and advertisement (0.8%; 2.89%) of water (Supporting Information: Appendix 4, Table S3). No other statistically significant changes were found. The sensitivity analysis produced similar results, when the sample was restricted to facilities where there was no nudge at baseline and the nudge of limited sugary drinks was implemented at follow‐up (Supporting Information: Appendix 4, Table S4).

#### 3.2.3 Health‐Related Knowledge and Water Consumption

More respondents identified that drinking sugary drinks leads to weight gain (baseline: 91.7%; follow‐up: 94.2%; change +2.5 pp. [95% CI +0.4, +4.6]) and increases the risk of heart disease (74.0%; 79.4%; change +5.4 pp. [95% CI +2.0, +8.9]) at follow‐up compared to baseline (Supporting Information: Appendix 4, Table S3). Self‐reported water consumption at the facilities (including purchased water and water from a facility tap or brought from home) was high at baseline (81.2%), with no difference at follow‐up. No change in water consumption was found in sensitivity analyses (Supporting Information: Appendix 4, Table S4).

### 3.3 Staff Survey

#### 3.3.1 Staff Demographic Characteristics

The staff survey was fully completed by 162 staff at baseline and 183 staff at follow‐up (20 staff were known to complete surveys at both time points) (Supporting Information: Appendix 4, Table S5). Six surveys were excluded from the analysis as respondents were not employed at facilities that were actively involved in the WIS intervention. At both baseline and follow‐up, most staff responding were employed by LG and worked in sport and recreation in a customer service role and had worked at that LG for more than 2 years. Staff who completed the survey at follow‐up were more likely to have been involved in making a healthy retail change (baseline: 35.7%, follow‐up: 53.8%).

#### 3.3.2 Staff Knowledge Regarding Organisational Priorities and Policies, and Support for Change

Staff perception of their organisation's intention to improve the healthiness of drinks available for sale in its sport and recreation facilities increased between baseline and follow‐up. Compared to baseline, at follow‐up, more staff reported that their organisation had made changes in the past 6 months that were still in place (baseline: 15.5%; follow‐up: 44.4%; *p* < 0.001) (Supporting Information: Appendix 4, Table S6).

A higher proportion of staff were aware of official policies relating to the provision of drinks within their LG's sport and recreation facilities at follow‐up, including reducing the amount of sugary drinks for sale (baseline: 29.8%; follow‐up: 58.9), no advertising of sugary drinks (16.4%; 33.7%) and increasing the availability of water (27.5%; 50.0%) (all *p* < 0.001) (Supporting Information: Appendix 4, Table S6).

At both baseline and follow‐up, agreement was high among staff that ‘sport and recreation centres have a responsibility to promote healthy eating’ (baseline: 82.8% agreed or strongly agreed; follow‐up: 78.2%; *p* = 0.857) and ‘removing sugary drinks from sport and recreation facility would lead to reduced consumption in the community’ (baseline: 61.1% agreed or strongly agreed; follow‐up: 59.0%; *p* = 0.902). There was no change in staff support for new or additional healthy retail changes (Supporting Information: Appendix 4, Table S6). Similar results were seen in the sensitivity analyses (Supporting Information: Appendix 4, Table S7).

## 4 Discussion

This study found that at baseline, the eight LGs involved in the WIS capacity‐building intervention reported a higher priority for promoting healthy eating and drinking, and improving the availability of healthy food and drink in their sport and recreation centres than 24 control LGs. Unsurprisingly, given the high priority for intervention LGs at baseline, priorities did not change for either group between baseline and follow‐up. Importantly, 50% of intervention LGs and only 17% of control LGs had official policies relating to healthy drinks provision at the end of the intervention period. A directly comparable survey question was not included at baseline.

Among customers in intervention LGs, increases were observed in the proportion of customers reporting awareness of the intervention between baseline and follow‐up; however, overall customer awareness remained low. Both customers and staff strongly supported healthy retail changes at both baseline and follow‐up.

### 4.1 Changes in Local Government Actions, Policies and Capacity for Change

The LG surveys demonstrated that LG priorities related to obesity prevention and healthy eating were higher in intervention LGs than in control LGs at baseline (including reducing the availability of sugary drinks in sporting facilities). Our previous impact evaluation of the WIS intervention [21] found that healthy changes were made to drinks displays in all eight intervention LGs, with all but one of 44 evaluated facilities demonstrating improvements in the healthiness of drinks available. The volume of least healthy drinks purchased decreased in both seasonal (−19.0 pp. [95% CI −28.6, −9.51]) and non‐seasonal (−11.0 pp. [95% CI −21.6, −0.41]) facilities. Combined with the results of the current study, this demonstrates that even when baseline priorities for promoting healthy eating are high, there is still an opportunity to support LG actions to improve the healthiness of the food environment and support healthier customer purchasing behaviour.

The fact that priorities in intervention LGs did not change between baseline and follow‐up reflects a strong ceiling effect (little scope for any increase) and may reflect that high council priority for change is a pre‐requisite for policy and practice progress. It was encouraging to see control LGs' priorities for obesity prevention and healthy eating did increase between baseline and follow‐up from a much lower base. That increase may be reflective of current work by Victorian state government and health promotion organisations to improve the food and drink environment in sport and recreation facilities. All Victorian LGs are subject to the same state funding and policy conditions. At the time of the study this included the ‘Healthy Choices: policy guidelines for sport and recreation centres’ [20], infrastructure funding incentives for sports and recreation facilities that implemented these guidelines [25] and a requirement to consider healthy eating as a priority area in councils' Municipal Public Health and Wellbeing Plans [26]. Variation in priorities between Victorian LGs indicates that LG‐level drivers may reflect local drivers such as support from internal and external stakeholders, level of control over facilities, internal and external funding availability, and facility and supplier contracts [10]. Public health efforts to address known barriers to change at LGs with low baseline support may help increase LG prioritisation of healthy eating and thereby increase uptake of healthy food policies.

### 4.2 Changes in Stakeholder Attitudes and Awareness

Three‐quarters of customers and staff surveyed at facilities within intervention LGs believed that sport and recreation facilities should promote healthy eating. Strong customer support for healthy change has been demonstrated in similar settings within Australia [4, 27] and internationally [17, 18]. A 2019 systematic scoping review of business outcomes of healthy food retail strategies reported that the majority of 50 studies reporting on customer perceptions found favourable perceptions towards implemented healthy retail interventions, including the introduction of healthy products and healthy food promotion [12]. Within the sporting setting, a systematic review of parent's and children's views on the sport‐related food environment [17] found no studies reported directly on children's perceptions of what should be available in sporting facilities. Three peer‐reviewed studies reported high pre‐intervention parental support for future changes and an acknowledgment of the dissonant messages in these settings due to their simultaneous promotion of physical activity and unhealthy food environments [17].

This is one of the first studies to examine changes in food policy stakeholder perspectives over time [4] and provides evidence that exposure may increase some elements of acceptance. This may spark a virtuous cycle whereby more favourable stakeholder perspectives encourage further healthy changes, leading to increased acceptance and more ambitious healthy changes [28]. It remains unclear what the relationship is between customer acceptance and purchasing and consumption of healthier foods. In the WIS intervention, both customers and staff had little confidence that removing sugary drinks would reduce consumption of these items. However, our evaluation of the impact of the WIS intervention showed that sales of sugary drinks did decline over time [21]. In line with self‐reported customer survey responses, there was no change in water sales [21]. Coupling ‘priming’ availability‐based interventions, such as reducing the range of unhealthy drinks, with other strategies, such as ‘salience’ interventions promoting healthier options, may increase their effectiveness [29]. Future studies including comparisons of staff and customer acceptance in control facilities would also help to separate consumer trends from intervention effects.

### 4.3 Strengths and Limitations

This study was strengthened by its multistakeholder perspective, recognising the importance of different groups for successful adoption, implementation and effectiveness of healthy food retail changes [28]. The pre‐post design advances the previous literature on the effect of healthy food retail changes on stakeholder customer and staff perspectives, which have predominantly included surveys only pre‐ or post‐intervention to date [17]. The non‐randomised repeat cross‐sectional design (and uncontrolled design for staff and customer surveys) means we were unable to account for the effect of the current momentum for change in healthy food retail in both sports and recreation [19] and LG settings [9], staffing changes, or for the priorities and characteristics of the small number of LGs and facilities voluntarily participating in such initiatives. Participating intervention and control LGs were similar in socioeconomic disadvantage, population size and rurality; however, the higher priorities for healthy eating in intervention LGs at baseline likely reflect a selection bias related to their application to participate in the intensive intervention. Although these factors reduce the external validity of the results, as one of the first studies to examine the change in stakeholder perspectives after exposure to a healthy food retail initiative in sport and recreation settings, it does provide important new lessons for LGs and health promotion researchers and advocates. Larger survey sample sizes may allow examination of whether LG responses differed by the extent of involvement, including dedicated staff load and number of participating facilities. Qualitative research may improve understanding of how to increase LG readiness for change and ultimately improve healthy food policy uptake.

## 5 Conclusion

Our study suggests that even where LGs already have a high priority for the provision of healthy drinks in their sports and recreation facilities, human resources and training interventions are an important stimulus for formal policy development and do lead to improvements in food environments and customer purchasing. Before and after a healthy drinks capacity‐building intervention, most customers and staff believed that sporting facilities should promote healthy eating. Even though they had doubts about whether removing sugary drinks would result in behaviour change, the change in purchasing behaviour seen in this intervention suggests that healthy retail food environment nudges in this setting are effective. More work is needed to align the healthy lifestyle message promoted by these facilities with the healthiness of their retail food environments and further increase the confidence of customers and staff in healthy retail interventions.

## Conflicts of Interest

The authors declare no conflicts of interest.

## Supporting information


Data S1.

## Data Availability

The data that support the findings of this study are available on request from the corresponding author. The data are not publicly available due to privacy or ethical restrictions.
