# Supplementary material for: Changes in Local Government Priorities and Stakeholder Satisfaction Following a Healthy Drinks Capacity‐Building Intervention in Sports and Recreation Facilities in Victoria, Australia
Source: Health Promot J Austr. 2025 Feb 24;36(2):e70024. doi: 10.1002/hpja.70024 (PMC11850953; doi:10.1002/hpja.70024)
Supplement: Supplementary file 1 — Data S1. [file HPJA-36-0-s001.docx]

**Supplemtary material to: Changes in local government priorities and stakeholder satisfaction following a healthy drinks capacity-building intervention in sports and recreation facilities in Victoria, Australia**

# **Appendix 1: Local government surveys**

#### Local government baseline survey

The following information is optional- and will be useful when we follow up in 2 years. Please note that your private information will not be made public at any stage.

**Organisation:**

**Name:**

**Email address:**

**Phone number:**

1. Which organisation are you representing?
2. Which of these best describes your position?
   - - - 1. Employed by the organisation in senior management
         2. Employed by the organisation in health promotion
         3. Other (please specify)__________________
3. Time in role:
   - - - 1. Less than 6 months
         2. 6 months to 1 year
         3. More than 1 year and less than 2 years
         4. 2 years or more

**Please answer each of the following questions to the best of your knowledge. The word ‘drinks’ refers to all non-alcoholic drinks and the word council refers to local government.**

1. Which of the following council owned **sports and recreation facilities that sell food or drinks** (e.g. through vending machines, kiosk and/or cafe but excluding once-off events like sausage sizzles or chocolate drives) are present in your local government area (LGA)?

Please specify the number of facilities. If unsure or you don't know please write "Don't Know"

**Please note:**If you have a facility that combines multiple categories (e.g. aquatic centre with outdoor hard courts) please add this to the “other (please describe)” category and provide the details of the combination.

|  | **Facilities that sell food/drink** | **Number of facilities** |
| --- | --- | --- |
| **Indoor sports stadium** |  |  |
| **Outdoor hard courts (e.g. netball, basketball, tennis)** |  |  |
| **Aquatic centres** |  |  |
| **Golf course** |  |  |
| **Gym** |  |  |
| **Ovals** |  |  |
| **Club facilities (please describe)** |  |  |
| **Other (please specify)** |  |  |
| **Other (please specific)** |  |  |

**Definition of sugary drinks:** sugary drinks refers to any drink that has added calories from sweeteners including soft drinks, sodas, sports and energy drinks, fruit drinks and flavoured milk. (Note, excludes all diet versions)

**Definition of diet drinks:**diet drinks refers to drinks that are sugar free, artificially sweetened and includes both diet and zero versions (e.g. PowerAde zero, diet coke).

1. Has your LGA implemented changes to improve the **healthiness of food and drinks available** in council owned sport and recreation facilities?
   1. Yes (only drinks)
   2. Yes (only food)
   3. Yes (food and drinks)
   4. No changes made
2. Which option below best describes **official policy** relating to the provision of non-alcoholic drinks within your organisation managed sport and recreation facilities?

**If you select 'yes' for the presence of a policy please provide more details about the policy for example** “our organisation implemented a policy that all of our aquatic centre cafés remove sugary drinks from display” and to the best of your knowledge please specify what year the change occurred and the number of facilities the change applies to.

**I am not aware of any policy that relates to drink provisions in sport and recreation facilities**

(If you have selected true please leave the table below blank)

- 1. True
  2. False

|  | **Yes** | **No** | **Unsure** | **Details about the policy** |
| --- | --- | --- | --- | --- |
| **No sugary drinks allowed to be sold** |  |  |  |  |
| **Sugary drinks must be hidden from customer (off display)** |  |  |  |  |
| **Reduced the amount of sugary drinks available for sale** |  |  |  |  |
| **No advertising of sugary drinks** |  |  |  |  |
| **Decrease the price of water** |  |  |  |  |
| **Increase availability of water** |  |  |  |  |
| **Other (please describe below)** |  |  |  |  |

1. The previous question asked about change related to official policy, this question relates to changes that may have occurred in the absences of official policy.

Which option below best describes health-promoting practices (relating to drink provisions) that any facilities in your local government area have implemented in **the absence of official policy?**

**If you select 'yes' for the presence of a change;**to the best of your knowledge please specify what year the change occurred and the number of facilities the change applies too.

**I am not aware that any facilities have made the changes below without a policy**

(If you have selected true please leave the table below blank)

- 1. True
  2. False

|  | **Yes** | **No** | **Unsure** | **Details about the policy** |
| --- | --- | --- | --- | --- |
| **No sugary drinks allowed to be sold** |  |  |  |  |
| **Sugary drinks must be hidden from customer (off display)** |  |  |  |  |
| **Reduced the amount of sugary drinks available for sale** |  |  |  |  |
| **No advertising of sugary drinks** |  |  |  |  |
| **Decrease the price of water** |  |  |  |  |
| **Increase availability of water** |  |  |  |  |
| **Other (please describe below)** |  |  |  |  |

1. Has your organisation engaged with any external organisations/individuals to assist with changing the food/drink environment in your facilities? *(For example: Healthy Eating Advisory Service, Dietitians)*
   1. Not applicable, since no changes were made
   2. No, we made the changes ourselves
   3. Yes –please specify

______________________________________________________________________________________________________________________________

1. Have you received**funding and/or in-kind support** to assist with changing the food and/or drink environment in your facilities?

If you have selected **'yes'** please specify if this was provided for food/drink/both and the support provided

- 1. Not applicable, since no changes were made
  2. No, we made the changes without funding
  3. Yes (please specify funding source________________________________________)

1. Which of the following does council think are**barriers** to making the food and/or drink environment healthier in sports and recreation facilities throughout your LGA?

(Please rank these from 1 to 7 where 1=most important and 7=least important)

If you think that only some of these are barriers, rank them accordingly (e.g. if you identify four barriers, label them from 1-4 and leave others as 0)

[ ] Inadequate funding

[ ] Inadequate support from key stakeholders (e.g. council members, centre staff, customer etc.)

[ ] Inadequate time

[ ] Inadequate control over facilities

[ ] Inability to source appropriate healthy alternatives (e.g. healthier drink options)

[ ] Problems negotiating with suppliers

[ ] other (please specify______________________________________)

1. Which of the following does council see as **facilitators**to making the food and/or drink environment healthier in sports and recreation facilities throughout your LGA?

(Please rank these from 1 to 7 where 1=most important and 7=least important)

If you think that only some of these are facilitators, rank them accordingly (e.g. if you identify four facilitators, label them from 1-4 and leave others as 0)

[ ] Adequate funding

[ ] Adequate support from key stakeholders (e.g. council members, centre staff, customer etc.)

[ ] Adequate time

[ ] Adequate control over facilities

[ ] Ability to source appropriate healthy alternatives (e.g. healthier drink options)

[ ] No issues negotiating with suppliers

[ ] other (please specify____________________________________)

1. Compared to one year ago, would you say the priority given to promoting healthy eating/drinking by the council has:
   1. Decreased
   2. Stayed the same
   3. Increased
2. Which of the following best represents council’s intentions to improve the healthiness of drink offerings available for sale in sport and recreation facilities throughout your LGA?
   1. Council has not thought about it
   2. Council is thinking about it
   3. Council is in preparation (planning programs and/or taking some steps)
   4. Council has made changes to the healthiness of drink offerings within the past 6 months
   5. Council has made changes to the healthiness of drink offerings more than 6 months ago which are still in place
   6. Council has made changes to the healthiness of drink offerings more than 6 months ago which are no longer in place

**For the following four questions please drag the bar across the line to the spot which most accurately represents councils current position**

1. Within your LGA would you say **promoting healthy eating/drinking** is a: (0= low priority, 10= high priority)

|  |  |  |  |  |  |  |  |  |  |
| --- | --- | --- | --- | --- | --- | --- | --- | --- | --- |
| **0** |  |  |  |  | **5** |  |  |  | **10** |
| Low priority | |  |  |  |  |  |  | High priority | |

1. What is the council's position on taking action to r**educe the prevalence of obesity in your LGA?** (0= we have not thought about it, 10=  it is a major focus)

|  |  |  |  |  |  |  |  |  |  |
| --- | --- | --- | --- | --- | --- | --- | --- | --- | --- |
| **0** |  |  |  |  | **5** |  |  |  | **10** |
| We have not thought about it | |  |  |  |  |  |  | We are giving it all our focus | |

1. What is the council's position on taking action to **increase the availability of healthy food and/or drink** in your council-owned sport and recreation facilities? (0= we have not thought about it, 10= We have completed all changes to increase availability of  healthy offerings)

|  |  |  |  |  |  |  |  |  |  |
| --- | --- | --- | --- | --- | --- | --- | --- | --- | --- |
| **0** |  |  |  |  | **5** |  |  |  | **10** |
| We have not thought about it | |  |  |  |  |  |  | We have completed all changes to increase availability of healthy offerings | |

1. What is the council's position on taking action to **reduce the availability of sugary drinks** for sale in your council-owned sport and recreation facilities? (0= we have thought about it, 10= we have fully removed sugary drinks)

|  |  |  |  |  |  |  |  |  |  |
| --- | --- | --- | --- | --- | --- | --- | --- | --- | --- |
| **0** |  |  |  |  | **5** |  |  |  | **10** |
| We have not thought about it | |  |  |  |  |  |  | We have fully removed sugary drinks | |

Please attach any official **council policies** relating to the provision of food and drinks within any of your council’s sport and recreation facilities? (optional)

If you need to upload multiple files please compress all files into a ZIP file before uploading

Files uploaded can be up to 100MB. For security reasons, executable files (such as those ending in .exe) are not permitted.

Or please enter the weblink below

_______________________________________________

Does the council have any **other**official policies relating to healthy food and drink provision within the community outside the sport and recreation scope?

For example: all council events must have free water available

- 1. Yes
  2. No
  3. I don’t know

Please attach any official **council policies** relating to healthy food and drink provision within the community**outside the** sport and recreation scope? (optional)

If you need to upload multiple files please compress all files into a ZIP file before uploading

Files uploaded can be up to 100MB. For security reasons, executable files (such as those ending in .exe) are not permitted.

Or please enter the weblink below

_____________________________________________________________________

Do you have any other comments you would like to make about health promotion practises throughout your LGA, including how VicHealth and the Department of Health and Human Services can help you achieve your goals in this area?

____________________________________________________________________________________________________________________________________________________

Do you have any other comment you would like to make regarding the survey?

______________________________________________________________________________________________________________________________________________________________________________________________________________________________

#### Local government follow-up survey

1. Which Local Government Area (LGA) are you representing?
2. In which state/ territory is your LGA located?
   1. New South Wales
   2. Victoria
   3. Queensland
   4. Western Australia
   5. South Australia
   6. Tasmania
   7. Australian Capital territory
   8. Northern Territory
3. Which of these bests describes your position?
   1. Employed by council in health promotion role
   2. Employed by council in sport and recreation role
   3. Employed by council as an environmental health officer
   4. Employed by council in community development or planning
   5. Other (please specify):

______________________________________________________

1. Time in role:
   1. Less than 6 months
   2. 6 months to 1 year
   3. More than 1 year and less than 2 years
   4. 2 years or more

**Please answer each of the following questions to the best of your knowledge. The word ‘drinks’ refers to all non-alcoholic drinks and the word ‘council’ refers to local government.**

1. Does your council own any sport or recreation facilities?
   1. Yes
   2. No

**Skip Logic**

1. Which of the following council-owned **sport and recreation facilities sell food or drinks** in your LGA?

Examples include: through vending machines, ice cream freezers, kiosk and/or café.

Note this excludes one-off events like sausage sizzles or chocolate fundraisers).

Please specify the number of facilities. If unsure or you don't know please write "Don't Know"

**Please note:** If you have a facility that combines multiple categories (e.g. aquatic centre with outdoor hard courts) please add this to the “other (please describe)” category and provide the details of the combination.

|  | **Facilities that sell food/drink (we have this facility that sells food or drinks; we have this facility, but it doesn’t sell food or drink; we don’t have this type of facility;**  **unsure)** | **Number of council- owned and managed facilities (enter 0 if not applicable)** | **Number of council- owned and externally managed facilities (enter 0 if not applicable)** |
| --- | --- | --- | --- |
| **Indoor sports**  **stadiums** |  |  |  |
| **Outdoor hard courts (e.g. netball,**  **basketball, tennis)** |  |  |  |
| **Aquatic centres** |  |  |  |
| **Golf courses** |  |  |  |
| **Gyms** |  |  |  |
| **Ovals** |  |  |  |
| **Club facilities**  **(please describe)** |  |  |  |
| **Other (please**  **specify)** |  |  |  |

**Please consult the definitions below which will be useful for the remaining questions.**

**Sugary drinks:** sugary drinks refer to any non-alcoholic water-based beverages with added sugar, including sugar-sweetened soft drinks and flavoured mineral waters, fortified waters, energy and electrolyte drinks, fruit and vegetable drinks, and cordials. This does not include fruit juice that is 100% fruit.

**Diet drinks:** diet drinks refers to drinks that are sugar free, sweetened with intense sweeteners (artificial or natural) and includes both diet and zero versions (e.g. PowerAde zero, diet coke).

**Healthy change to drinks**: any changes made to the drinks available or promoted such as reducing the amount of sugary drinks available, increasing the availability of healthy options such as water, 100% fruit juice and small flavoured milks, or decreasing marketing of unhealthy drinks.

**Healthy changes to food:** any changes made to the food available and promoted that results in reduced unhealthy food options and increased healthy food options. This includes reducing the availability of deep-fried options and/or high fat and sugar snacks (such as chocolate and ice cream), reducing marketing of unhealthy foods and increasing the variety of whole grains or fruit or vegetable products.

1. Are you aware of any written council policy that relates to healthiness of food and/or drinks available in sport and recreation facilities?
   1. Yes (only drinks)
   2. Yes (only food)
   3. Yes (food and drinks)
   4. No policy relating to food or drinks

**Skip Logic**

1. Which of the options below are part of **official council policy** relating to the provision of food and non-alcoholic drinks within your council-owned sport and recreation facilities?

**If you select 'yes' for the presence of a policy please provide more details about the policy and to the best of your knowledge please specify what year the change occurred (policy was implemented) and the number of facilities to which the change applies.** For example “In 2017 our council implemented a policy that all of our aquatic centre cafés remove sugary drinks from display (placed behind counter or covered with poster in fridge)".

|  | **Presence of policy in your LGA** | | |  | | |
| --- | --- | --- | --- | --- | --- | --- |
|  | **Yes** | **No** | **Unsure** | **Details about the policy** | **Year policy adopted** | **Number of facilities that have begun implementing the policy to**  **date** |
| **Drink changes:** | | | | | | |
| **No sugary drinks**  **allowed to be sold** |  |  |  |  |  |  |
| **Sugary drinks must be hidden from customer (off display)** |  |  |  |  |  |  |
| **Reduced the display of sugary drinks** |  |  |  |  |  |  |
| **Reduced the amount/range of sugary drinks**  **available for sale** |  |  |  |  |  |  |
| **No advertising or**  **promotion of sugary drinks** |  |  |  |  |  |  |
| **Decreased the**  **price of water** |  |  |  |  |  |  |
| **Increased the availability of water (free and/or for**  **purchase)** |  |  |  |  |  |  |
| **Labelling drinks options using a**  **traffic light labelling system** |  |  |  |  |  |  |
| **Other (please describe)** |  |  |  |  |  |  |
| **Food changes:** | | | | | | |
| **Increase the prominence or display of healthy food**  **options** |  |  |  |  |  |  |
| **Decreased the prominence or display or unhealthy food**  **options** |  |  |  |  |  |  |
| **Increased number of healthy food**  **options** |  |  |  |  |  |  |
| **Decreased the number of unhealthy**  **food options** |  |  |  |  |  |  |
| **No value deals with unhealthy options (e.g. free soft drink with sandwich or free chips with a**  **burger)** |  |  |  |  |  |  |
| **Labelling food options using a traffic light labelling**  **system** |  |  |  |  |  |  |
| **No advertising or promotion**  **of unhealthy foods** |  |  |  |  |  |  |
| **Other (please**  **describe)** |  |  |  |  |  |  |

1. The previous question asked about changes related to official council policy, this question relates to changes that may have occurred in the **absence of official policy**.

Has your LGA made changes to improve the **healthiness of food and/or drinks available** in council-owned sport and recreation facilities in the **absence of official policy**?

- 1. Yes (only drinks)
  2. Yes (only food)
  3. Yes (food and drinks)
  4. No changes made in the absence of official policy

**Skip logic**

**If you select 'yes' for the presence of a change;** to the best of your knowledge please specify what year the change was implemented and the number of facilities the change applies too.

|  | **Did the change occur?** | | |  | | |
| --- | --- | --- | --- | --- | --- | --- |
|  | **Yes** | **No** | **Unsure** | **Details about the change** | **Year begun** | **Number of facilities where change has been**  **implemented** |
| **Drink changes:** | | | | | | |
| **No sugary drinks**  **allowed to be sold** |  |  |  |  |  |  |
| **Sugary drinks must be hidden from customer (off display)** |  |  |  |  |  |  |
| **Reduced the display of sugary drinks** |  |  |  |  |  |  |
| **Reduced the amount of sugary drinks available**  **for sale** |  |  |  |  |  |  |
| **No advertising or promotion of**  **sugary drinks** |  |  |  |  |  |  |
| **Decreased the**  **price of water** |  |  |  |  |  |  |
| **Increased the availability of water (free and/or**  **for purchase)** |  |  |  |  |  |  |
| **Labelling drinks options using a traffic light**  **labelling system** |  |  |  |  |  |  |
| **Other (please describe)** |  |  |  |  |  |  |
| **Food changes:** | | | | | | |
| **Increase the prominence or display of healthy food**  **options** |  |  |  |  |  |  |
| **Decreased the prominence or display or unhealthy**  **food options** |  |  |  |  |  |  |
| **Increased number of healthy food**  **options** |  |  |  |  |  |  |
| **Decreased the number of unhealthy**  **food options** |  |  |  |  |  |  |
| **No value deals with unhealthy options (e.g. free soft drink with sandwich or free chips with a**  **burger)** |  |  |  |  |  |  |
| **Labelling food options using a traffic light labelling**  **system** |  |  |  |  |  |  |
| **No advertising or promotion of unhealthy**  **foods** |  |  |  |  |  |  |
| **Other**  **(please describe)** |  |  |  |  |  |  |

1. Has your council engaged with any other organisations or individuals to assist with changing the food and/or drink environment in council-owned sport and recreation facilities?
2. No, we made the changes ourselves
3. Yes –please specify
4. Has council received **funding and/or in-kind support** to assist with changing the food and/or drink environment in your facilities?
   1. Not applicable, since no changes were made
   2. No, we made the changes without funding and/or in-kind support
   3. Yes, we made the changes with funding and/or in-kind support

If yes:

What was the funding and/or in-kind support targeted at?

1. Food
2. Drinks
3. Both

Please provide details of the type of support that was provided

***End Skip logic***

1. Which of the following does **council leadership** see as **barriers** to making the food and/or drink environment healthier in sport and recreation facilities throughout your LGA?

(Please rank these from 1 to 10 where 1=most important and 10=least important)

If you think that only some of these are barriers, rank them accordingly (e.g. if you identify four barriers, label them from 1-4 and leave others as 0)

[ ] Inadequate funding (this can include lack of funding to have a dedicated employee)

[ ] Inadequate support from internal stakeholders (e.g. council elected members, council staff, centre staff)

[ ] Inadequate support from external stakeholders (e.g. customers, community members)

[ ] Inadequate staff time

[ ] Inadequate control over facilities (e.g. council doesn’t mange the kiosk)

[ ] Inability to source appropriate healthy alternatives (e.g. healthier drink options)

[ ] Problems negotiating with suppliers

[ ] Concerns relating to impact on financial viability of food outlet(s)

[ ] Lack of healthy food and drink policy

[ ] Other (please specify )

1. Which of the following does **council leadership** see as the most important **facilitators** to making the food and/or drink environment healthier in sport and recreation facilities throughout your LGA?

(Please rank these from 1 to 10 where 1=most important and 10 =least important)

If you think that only some of these are facilitators, rank them accordingly (e.g. if you identify four facilitators, label them from 1-4 and leave others as 0)

[ ] Adequate funding (e.g. ability to hire a person for this role)

[ ] Adequate support from internal stakeholders (e.g. council elected members, council staff, centre staff)

[ ] Adequate support from external stakeholders (e.g. customers, community members)

[ ] Adequate time

[ ] Adequate control over facilities

[ ] Ability to source appropriate healthy alternatives (e.g. healthier drink options)

[ ] Suppliers who are easy to negotiate with

[ ] Financial viability of food outlet not a concern

[ ] Presence of healthy food and drink policy

[ ] Other (please specify )

1. Which of the following best represents council’s intentions to improve the healthiness of

drink offerings available for sale in sport and recreation facilities throughout your LGA?

1. Council has not yet seriously thought about making changes to drink offerings
2. Council is thinking about it but have not begun any other preparation for making changes to drink offerings
3. Council is currently preparing to make changes to the drink offerings (planning programs and/or taking some steps)
4. Council has made changes to the healthiness of drink offerings within the past 6 months
5. Council has made changes to the healthiness of drink offerings **more than 6 months ago,** which are **still fully in place**
6. Council has made changes to the healthiness of drink offerings **more than 6 months ago**, which are **no longer fully in place**
7. Which of the following best represents council’s intentions to improve the healthiness of

food offerings available for sale in sport and recreation facilities throughout your LGA?

1. Council has not yet seriously thought about making changes to food offerings
2. Council is thinking about it but have not begun any other preparation for making changes to food offerings
3. Council is currently preparing to make changes to the food offerings (planning programs and/or taking some steps)
4. Council has made changes to the healthiness of food offerings within the past 6 months
5. Council has made changes to the healthiness of food offerings **more than 6 months ago,** which are **still fully in place**
6. Council has made changes to the healthiness of food offerings **more than 6 months ago**, which are **no longer fully in place**

***End Skip logic***

1. Compared to one year ago, would you say the priority given to promoting healthy eating/drinking by the council has:
2. Decreased
3. Stayed the same
4. Increased
5. Unsure
6. Has your council collaborated with any local health services to develop and/or support local promotion of healthy eating and drinks?
7. No
8. Yes –please report details of the collaboration, including financial and other support provided.

**For the following four questions please drag the bar across the line to the spot which most accurately represents council leadership’s current position**

1. Within your LGA would you say **promoting healthy eating/drinking** is a: (0= low priority, 5= medium priority, 10= high priority)

| **0** | **5** | **10** |
| --- | --- | --- |
| Low priority |  | High priority |

1. What is the council's position on taking action to **reduce the prevalence of obesity in your LGA?** (0= we have not thought about it, 10= it is a major focus)

| **0** | **5** | **10** |
| --- | --- | --- |
| We have not thought  about it |  | We are  giving it all our focus |

1. What is the council's position on taking action to **improve public health and wellbeing LGA?** (0= we have not thought about it, 10= it is a major focus)

| **0** | **5** | **10** |
| --- | --- | --- |
| We have not thought  about it |  | We are  giving it all our focus |

**Skip Logic* Only local governments that noted change**

1. What is the council's position on taking action to **increase the availability of healthy food and/or drink** in your council-owned sport and recreation facilities? (0= we have not thought about it, 10= We have completed all changes to increase availability of healthy offerings)

| **0** | **5** | **10** |
| --- | --- | --- |
| We have not thought  about it |  | We have completed  all changes to increase availability of healthy  offerings |

1. What is the council's position on taking action to **reduce the availability of sugary drinks for sale** in your council-owned sport and recreation facilities? (0= we have not thought about it, 10= we have fully removed sugary drinks)

| **0** | **5** | **10** |
| --- | --- | --- |
| We have not thought  about it |  | We have fully  removed sugary drinks |

1. What is the council's position on taking action to **reduce the availability of unhealthy foods for sale** in your council-owned sport and recreation facilities? (0= we have not thought about it, 10= we have fully removed unhealthy food)

| **0** | **5** | **10** |
| --- | --- | --- |
| We have not thought  about it |  | We have fully  removed unhealthy food |

1. Please attach any official **council policies** relating to the provision of food and drinks within

any of your council’s sport and recreation facilities.

If you need to upload multiple files please compress all files into a ZIP file before uploading

Files uploaded can be up to 100MB. For security reasons, executable files (such as those ending in .exe) will not be opened.

Or please enter the weblink below

***End skip logic***

1. Does the council have any **other** official policies relating to healthy food and drink provision within the community outside of sport and recreation?

(This may include, for example, health care services, schools and childcare centres). For example: all council events must have free water available

- 1. Yes
  2. No
  3. I don’t know

1. Please attach any official **council policies** relating to healthy food and drink provision within the community **outside of sport and recreation** (optional)

For example: Healthy catering policy for staff events

If you need to upload multiple files please compress all files into a ZIP file before uploading. Files uploaded can be up to 100MB. For security reasons, executable files (such as those ending in .exe) will not be opened.

Or please enter the weblink below

1. Do you have any other comments you would like to make about public health practices throughout your LGA, including how the state government can help you achieve your goals in this area?
2. Do you have any other comments you would like to make regarding the survey?

# **Appendix 2: Customer surveys**

*[NB: changes between baseline and follow-up customer surveys are noted throughout in square backets]*

**You must be 15 years or older to complete this survey**

***Definition of sugary drinks:****sugary drinks refers to any drink that has added calories from sweeteners including soft drinks, sodas, sport and energy drinks, fruit drinks, flavoured milk excluding the diet version*

***Definition of diet drinks:*** *diet drinks refers to drinks that are sugar free, artificially sweetened and include the diet and zero versions (e.g. PowerAde zero, diet coke).*

1. **Please select which sport and recreation facilities you are at today?** (Please tick one)
2. **In the past 6 months, how often would you usually purchase food or drink from the food outlet at this facility?** (Please circle one)
3. Everyday
4. Three or more days a week
5. One or two days a week
6. Less than once a week
7. Less than twice per month
8. Never before

*[Questions 3 and 4 included in baseline survey only.]*

| 1. **What food/s did you purchase from the food outlet at this facility today? (Tick all that apply)**  - Lollies, chocolate, chips (packet) - Cakes, biscuits, muffins - Ice creams or icy poles - Sandwiches, rolls, wraps - Fruit, vegetables - Sausage rolls, hot chips, fried foods - Hot meals - Sushi - Other  Please specify: ________________________________ ________________________________ - I didn't purchase food | 1. **What drink/s did you purchase from the food outlet at this facility today?**   **(Tick all that apply)**   - Regular soft drink or regular energy drink - Diet soft drink or diet energy drink - Water - Slushy - Milkshake/thick shake - Smoothie - Coffee/ tea - Hot chocolate - Other Please specify: ________________________________ ________________________________ - I didn’t purchase drinks |
| --- | --- |

1. **Did you drink any water while you were at the facility today?**

- Yes
- No

1. **If yes, when you drank water did you** (tick all relevant)**:**

- purchase water at the facility
- bring water from home
- purchase water from outside the facility
- use the water fountain at the facility

*[Question 7 included in baseline survey only]*

1. **What would make you more likely to drink from the water fountain?** (tick all that apply)

- Cleanliness of fountain
- Appearance of the fountain
- Change in water temperature
- Better location
- Water pressure
- Other
  Please specify ______________________________________________________________
- I am happy with the water fountains
- No water fountains at this facility

1. **Did you consume any food or drinks while you were at the facility today that were brought from outside the facility?**

- Yes
- No

1. **If yes, what did you bring from outside the facility?** (Tick all relevant)

- Lollies, chocolate, chips (packet)
- Cakes, biscuits, muffins
- Ice creams or icy poles
- Sandwiches, rolls, wraps
- Fruit, vegetables
- Sausage rolls, hot chips, fried foods
- Hot meals
- Sushi
- Regular soft drink or regular energy drink
- Diet soft drink or diet energy drink
- Water
- Slushy
- Milkshake/thick shake
- Smoothie
- Coffee/ tea
- Hot chocolate
- Other
  Please specify ______________________________________________________________

***Definition of sugary drinks:****sugary drinks refers to any drink that has added calories from sweeteners including soft drinks, sodas, sport and energy drinks, fruit drinks, flavoured milk excluding the diet version*

***Definition of diet drinks:*** *diet drinks refers to drinks that are sugar free, artificially sweetened and include the diet and zero versions (e.g. PowerAde zero, diet coke).*

1. **Have you noticed any changes to the food and drink offered at the facility in the last 6 months?**

- Yes
- No

1. **If yes, what have you noticed?** (Tick all relevant)

- Changes in the variety of sugary drinks
- Changes to the availability of sugary drink
- Changes to fridge display
- Changes to prices
- Changes in the variety of water
- Changes to the availability of water
- Changes to advertising of water
- Other. Please specify: _________________________________________________________

1. **On how many days in the past week did you drink regular sugary drinks (non- diet version)?**

| None | 1-2 | 2-4 | 5-6 | 7 |
| --- | --- | --- | --- | --- |

1. **On how many days in the past week did you drink diet sugary drinks?**

| None | 1-2 | 2-4 | 5-6 | 7 |
| --- | --- | --- | --- | --- |

1. **Considering your current intake of sugary drinks, would you like to** (circle one)**:**

| Drink much less | drink less | drink the same amount | drink more | drink much more |
| --- | --- | --- | --- | --- |

1. **Where is the most common place that your purchase sugary drinks?**

- Supermarket
- Convenience store
- Restaurants
- Sport and recreation facilities
- Other. Please specify: _________________________________________________________
- I don’t purchase sugary drinks

1. **Which of the following do you think are healthy drink options for drinking every day?**

(Tick all that apply)

- Water
- Juice
- Milk
- Regular soft drinks/regular energy drinks
- Diet soft drinks/diet energy drinks

1. **Please select all those statements that apply. Drinking sugary drinks often will…**

- Lead you to gain weight
- Increase your risk of heart disease
- Have no impact on your health
- Increase your risk of diabetes
- Help you live a healthier life
- Increase your risk of dental decay

1. **Do you believe your community needs to implement changes to reduce sugary drink consumption? (Circle one)**

| Definitely yes | probably yes | probably not | definitely not | unsure |
| --- | --- | --- | --- | --- |

1. **Who do you think is responsible for reducing the consumption of sugary drinks?** (Tick all that apply)

- Me
- Local Government
- Federal Government
- Parents of children
- Primary and secondary schools
- Other. Please specify: _________________________________________________________
- I don't think the level of consumption needs to be changed
- I don’t know

1. **If sugary drinks were removed from sales from your sports facility would you be most likely to** (tick one)**:**

- Buy no drinks
- Buy another sort of drink
- Bring your own sugary drinks (diet and regular) from outside
- Not applicable - I don’t buy sugary drinks
- Other. Please specify: _________________________________________________________

1. **What do you think sport and recreation facilities should do to help reduce the consumption of sugary drinks?** (Tick all that apply)

- Increase the price of sugary drinks
- Reduce the price of water
- Remove all sugary drinks from customer view
- Remove all sugary drinks completely from sale
- Limit the amount of sugary drinks for sale
- Other. Please specify: _________________________________________________________
- I don’t think they should change anything

1. **In general, how much do you agree with the statement: “removing sugary drinks from sport and recreation facilities will lead to reduced consumption in the community”?** (Circle one)

| Strongly disagree | Disagree | Neither agree nor disagree | Agree | Strongly Agree |
| --- | --- | --- | --- | --- |

1. **How much do you agree with the statement: “Sport and recreation centres have a responsibility to promote healthy eating”?** (Circle one)

| Strongly disagree | Disagree | Neither agree nor disagree | Agree | Strongly Agree |
| --- | --- | --- | --- | --- |

1. **If you have any other feedback you would like to provide about this food outlet, please provide this below.**

___________________________________________________________________________

___________________________________________________________________________

___________________________________________________________________________

**About you (demographic questions):**

1. **Age** (circle one):

| 15 – 17 | 18 – 24 | 25 – 34 | 35 – 44 | 45 – 54 | 55 – 64 | 65 years or older |
| --- | --- | --- | --- | --- | --- | --- |

1. **Gender**  (tick one):

- Female
- Male
- Other

1. **What is your highest level of formal education that you have completed?**

- Still in high school
- Did not complete high school
- Year 12 or Trade certificate or diploma
- University degree or higher

1. **Postcode (home):** ___________

**About your visit today:**

1. **No. of adults in your group** (tick one)**:**

- 1
- 2
- 3 +

1. **No. of children your group** (tick one)**:**

- 0
- 1
- 2
- 3 +

# **Appendix 3: Local government and facility staff survey**

**You must be 18 years or over to complete this survey**

**Definition of sugary drinks:** sugary drinks refers to any drink that has added calories from sweeteners including soft drinks, sodas, sport and energy drinks, fruit drinks, flavoured milk excluding the diet version

**Definition of diet drinks:** diet drinks refers to drinks that are sugar free, artificially sweetened and include the diet and zero versions (e.g. PowerAde zero, diet coke).

1. **Please select which sport and recreation facility you work in (where applicable)**
2. **Which of these best describes your job position?**
   1. Employed by council in senior management
   2. Employed by council in health promotion
   3. Employed by council in sport and recreation
   4. Employed by sport and recreation facility in management role
   5. Employed by sport and recreation facility in customer service or front-of-house role
   6. Volunteer at sport and recreation facility in management role
   7. Volunteer at sport and recreation facility in customer service or front-of-house role
   8. Other (please specify)__________________
3. **Time in role:**
4. Less than 6 months
5. 6 months to 1 year
6. More than 1 year and less than 2 years
7. 2 years or more
8. **In the past 6 months, how often have you personally purchased food or drink from council owned sport and recreation centres**
   1. Every day
   2. Three or more days a week
   3. One or two days a week
   4. Less than once a week
   5. Less than twice per month
   6. Never before
9. **Within your organisation would you say promoting healthy eating is a:**
10. Low priority
11. Medium priority
12. High priority
13. **Compared to one year ago, would you say the priority given to promoting healthy eating within your organizations has:**
14. Decreased
15. Stayed the same
16. Increased
17. I don’t know
18. **Which of the following best represents your organisation’s intent to improve the healthiness of your drink offerings available for sale in your sport and recreation facilities?**
19. We have not thought about it
20. We are thinking about it
21. We are in preparation (planning programs and/or taking some steps)
22. We have made changes to the healthiness of drink offerings within the past 6 months
23. We made changes to the healthiness of drink offerings more than 6 months ago which are still fully in place
24. We made changes to the healthiness of drink offerings more than 6 months ago which are no longer fully in place

**Definition of sugary drinks:** sugary drinks refers to any drink that has added calories from sweeteners including soft drinks, sodas, sport and energy drinks, fruit drinks, flavoured milk excluding the diet version

1. **Are you aware of any official policies relating to the provision of drinks within your council’s sport and recreation facilities that include the following? (select as many that apply)**
2. No sugary drinks allowed to be sold
3. Sugary drinks must be hidden from customer (off display)
4. Reduced the amount of sugary drinks available for sale
5. No advertising of sugary drinks
6. Decrease the price of water
7. Increase availability of water
8. Other (please specify_____________________________________________)
9. I’m not sure
10. **Have you been involved with any healthy food or drinks changes made in your organisation?**
11. Yes
12. No
13. **If yes, what has been your role? (select all that apply)**
14. Implementing the changes (e.g. ordering stock, rearranging shelves)
15. Policy development (planning, writing, approving policy)
16. Talking to customers about changes
17. Other (please specify)
18. **Would you support your council making additional/new health related changes to your sport and recreation facilities related to the provision of healthy food and drinks?**
19. Yes
20. No

**Definition of sugary drinks:** sugary drinks refers to any drink that has added calories from sweeteners including soft drinks, sodas, sport and energy drinks, fruit drinks, flavoured milk excluding the diet version

1. **In general, how much do you agree with the statement “removing sugary drinks from sport and recreation facilities will lead to reduced consumption in the community?”**
2. Strongly disagree
3. Disagree
4. Neither agree nor disagree
5. Agree
6. Strongly Agree
7. **How much do you agree with the statement: “Sport and recreation centres have a responsibility to promote healthy eating.”**
   1. Strongly disagree
   2. Disagree
   3. Neither agree nor disagree
   4. Agree
   5. Strongly Agree
8. **Would you be willing to be contacted by the researchers for further follow-up concerning healthy drinks changes in sport and recreation facilities, for a:**
9. Follow up survey
10. Follow up interview
11. Neither
12. **If you have selected a or b in the question above, please provide your email address** __________________________

**Email addresses will only be available to [anonymised] university researchers involved in the project in order to look at changes in responses over time and/or to invite you to participate in further research for this project. Email addresses WILL NOT be used to identify you in any communication with your employer or in any publication.**

**Thank you for participating in this survey. Please add any final comments you have about the survey or this project: _____________________________________________________________________________________________________________________________________________________**

# **Appendix 4: Additional survey results**

###

### **Table S1: Comparison of change in local government priorities relating to promoting healthy eating and obesity in Water in Sport intervention versus control participating local governments (n = 32 local governments)**

| **Priority given in local government to** | **Mean [95% confidence interval]** | | | | | | |
| --- | --- | --- | --- | --- | --- | --- | --- |
|  | **Intervention councils (n = 8)** | | | **Control councils (n = 24)** | | | **Intervention effect (difference between intervention and control councils)** |
|  | **Baseline** | **Follow-up** | **Change** | **Baseline** | **Follow-up** | **Change** |  |
| Promoting healthy eating/drinking ^a^ | 8.6 [7.6,9.6] | 7.5 [6.4,8.5] | -1.1 [‑2.6,+0.3] | 6.5 [5.6,7.5] | 5.2 [4.3,6.1] | -1.4 [23.8,+0.1] | ‑0.3 [‑1.8,+2.3] |
| Reducing the prevalence of obesity ^b^ | 7.9 [6.0,9.7] | 7.5 [6.4,8.6] | -0.4 [‑2.7,+1.9] | 6.7 [5.7,7.7] | 6.6 [5.7,7.7] | -0.1 [‑1.6,+1.4] | ‑0.3 [‑3.0,+2.5] |
| Increasing the availability of healthy food/drink in LG owned sport and recreation facilities ^c^ | 8.5 [7.2,9.8] | 7.4 [6.2,8.5] | -1.1 [‑2.7,+0.5] | 4.9 [3.8,6.0] | 5.3[4.5,6.2] | +0.4[‑1.3,+2.1] | ‑1.5[‑3.9,+0.8] |
| Reducing the availability of sugary drinks for sale in LG owned sport and recreation facilities ^d^ | 8.1 [6.6,9.6] | 7.6 [6.9,8.3] | -0.5 [‑2.3,+1.3] | 4.9 [3.8,6.1] | 5.2 [4.3,6.2] | +0.3 [‑1.6,+2.2] | ‑0.8 [‑3.8,+6.1] |
| Improving public health and wellbeing ^e^ | N/A | 8.4[7.3,9.5] | N/A | N/A | 8.7[8.0,9.3] | N/A | N/A |

LG, Local Government

No differences were statistically significant (all p>0.05).

^a^ “Within your LG would you say promoting healthy eating/drinking is a: (rank priority) (11-point priority scale: 0= low priority, 10= high priority)?”

^b^ “What is your local government’s position on taking action to reduce the prevalence of obesity in your LG? (11-point priority scale: 0= we have not thought about it, 10= it is a major focus)”

^c^ “What is your local government’s position on taking action to increase the availability of healthy food/drink in LG owned sport and recreation facilities? (11-point priority scale: 0= we have not thought about it, 10= we have completed all changes to increase availability of healthy offerings)”

^d^ “What is the local government’s position on taking action to reduce the availability of sugary drinks for sale in your LG-owned sport and recreation facilities? (11-point priority scale: 0= we have thought about it, 10= we have fully removed sugary drinks)”

^e^ This question was not asked in the baseline survey.

### **Table S2: Characteristics of all local government customer respondents at baseline (2018, n = 1,079) and follow-up (2019, n = 1,188)**

| **Characteristic** | **n (%)** | |
| --- | --- | --- |
|  | **Baseline** | **Follow-up** |
| **Gender** | n = 1,040 | n = 1,165 |
| Male | 271 (26.1) | 298 (25.7) |
| Female | 767 (73.9) | 861 (74.2) |
| **Age (years)** | n = 1047 | n = 1166 |
| 15 to 24 | 84 (8.0) | 107 (9.2) |
| 25 to 44 | 459 (43.8) | 493 (42.3) |
| 45 to 64 | 363 (34.7) | 405 (34.7) |
| 65 and older | 141 (13.5) | 161 (13.8) |
| **Education** | n = 1,041 | n = 1,159 |
| Did not complete high school | 84 (8.7) | 88 (7.6) |
| Still in high school | 29 (2.8) | 36 (3.1) |
| Year 12 or trade certificate | 418 (40.2) | 478 (41.2) |
| University degree or higher | 510 (49.0) | 557 (48.1) |
| **SEIFA ^a^ quintiles** | n = 1,300 | n = 1,496 |
| 1 (most disadvantaged) | 399 (30.7) * | 407 (27.2) * |
| 2 | 202 (15.5) * | 415 (27.7) * |
| 3 | 589 (45.3) * | 470 (31.4) * |
| 4 | 119 (8.5) * | 204 (13.6) * |
| 5 (least disadvantaged) | 0 (0.0) * | 0 (0.0) * |
| **Purchase frequency from facility café** | n = 1145 | n = 1300 |
| Never | 408 (35.6) * | 481 (37.4) * |
| Less than once per week | 187 (16.3) * | 183 (14.1) * |
| 1-4 times per week | 537 (46.9) * | 612 (47.1) * |
| ≥ 5 times per week | 13 (1.1) * | 19 (1.5) * |

^a^ LGs are ranked from most disadvantaged (1) to least disadvantaged (10) using the decile rank within Australia. Australian Bureau of Statistics. Census of Population and Housing: Socio-Economic Indexes for Areas (SEIFA) 2016. [Internet]. Canberra, Australia. 2021. [cited 2021 July]. Available from: https://www.abs.gov.au/ausstats/abs@.nsf/mf/2033.0.55.001

* Statistically significant differences (p<0.05) between responses at baseline and at follow-up.

### **Table S3: Customer knowledge and attitudes in relation to water and sugary drinks, awareness of interventions and self-reported consumption of water at baseline in Water in Sport intervention local governments (2018, n = 1,079) and follow-up (2019, n = 1,118)**

| **Survey question and possible answers** | **Mean proportion respondents [95% CI] ^a^** | | **Percentage point difference in response between baseline and follow-up [95% CI]** |
| --- | --- | --- | --- |
|  | **Baseline (n = 1,079)** | **Follow-up (n =** **1,188)** |  |
| **Awareness of change: Have you noticed changes to the… ^b^:** |  |  |  |
| Food and drink offered at the facility | 21.1 [18.8,23.5] | 21.6 [19.3,23.8] | +0.5 [-2.8,+3.7] |
| Variety of sugary drinks | 4.0 [2.8,5.1] | 6.0 [4.6,7.4] | +2.0 [+0.2,+3.9] * |
| Availability of sugary drinks | 3.7 [2.6,4.8] | 7.8 [6.3,9.4] | +4.1 [+2.2,+6.1] * |
| Fridge display | 10.0 [8.3,11.8] | 13.1 [11.1,15.1] | +3.1 [+0.4,+5.7] * |
| Price of drinks | 3.9 [2.8,5.0] | 4.6 [3.4,5.8] | +0.7 [-0.9,+2.4] |
| Variety of water | 2.0 [1.2,2.9] | 3.5 [2.4,4.6] | +1.5 [+0.1,+2.8] * |
| Availability of water | 1.9 [1.1, 2.7] | 4.1 [2.9, 5.2] | +2.2 [+0.8,+3.6] * |
| Advertising of water | 0.8 [0.3,1.3] | 2.9 [1.9,3.9] | +2.1 [+1.0,+3.2] * |
| **Knowledge related questions ^b^:** |  |  |  |
| *Is the following a healthy drink for everyday:* |  |  |  |
| Water | 98.5 [97.8,99.3] | 98.6 [97.9,99.3] | +0.1 [-0.9,+1.0] |
| Juice | 1.3 [1.1,1.5] | 1.4 [1.2,1.6] | +0.1 [-1.2,+4.3] |
| Sugary drinks | 1.5 [0.8,2.3] | 1.0 [0.4,1.5] | -0.5 [-1.5,+0.4] |
| Diet sugary drinks | 2.7 [1.8,3.7] | 4.2 [3.1,5.3] | +1.5 [-0.0,+2.9] |
| *Drinking sugary drinks often will…* |  |  |  |
| Lead to weight gain | 91.7 [90.0,93.3] | 94.2 [92.8, 95.5] | +2.5 [+0.4,+4.6] * |
| Increase your risk of heart disease | 74.0 [71.4,76.6] | 79.4 [77.2,81.7] | +5.4 [+2.0,+8.9] * |
| Have no impact on your health | 9.0 [7.3,10.7] | 8.1 [6.5,9.6] | -0.9 [-3.2,+1.4] |
| Increase your risk of diabetes | 85.1 [83.0,87.2] | 85.1 [83.1,87.1] | +0.0 [-2.9,+2.9] |
| Help you live a healthier life | 2.5 [1.5,3.4] | 3.0 [2.0,3.9] | +0.5 [-0.8,+1.8] |
| Increase your risk of dental decay | 85.9 [83.9,88.0] | 85.0 [84.0,88.0] | -0.9 [-2.8,+2.8] |
| **Water consumption ^b^ :** |  |  |  |
| Drank water while at the facility on day of survey | 81.2 [78.9,83.5] | 81.1 [79.0,83.3] | -0.1 [-3.1,+3.0] |
| **Agreement with:** |  |  |  |
| *Sport and recreation facilities have a responsibility to promote healthy eating* | | | |
| Strongly disagree | 6.0 [4.9,7.0] | 6.0 [4.9,7.0] | 0.0 [-0.8,+0.9] |
| Disagree | 4.2 [3.3,5.1] | 4.2 [3.3, 5.1] | 0.0 [-0.5,+0.5] |
| Neither disagree no agree | 13.1 [11.5,11.5] | 13.1 [11.5,14.6] | 0.0 [-1.3,+1.3] |
| Agree | 37.0 [34.9,39.0] | 37.0 [35.0,39.0] | 0.0 [-0.9,+0.9] |
| Strongly agree | 39.9 [37.1,42.6] | 39.8 [37.2,42.5] | -0.1 [-3.7,+3.6] |
| *Removing sugary drinks from sport and recreation facilities will lead to reduced consumption on the community* | | | |
| Strongly disagree | 8.5 [7.2,9.7] | 9.4 [8.0,10.71] | +0.9 [-0.3,+2.1] |
| Disagree | 14.6 [13.0,16.3] | 15.7 [14.0,17.4] | +1.1 [-0.4,+2.6] |
| Neither disagree no agree | 22.6 [20.8,24.4] | 23.4 [21.6,25.2] | +0.7 [-0.3,+1.7] |
| Agree | 35.9 [33.8,38.1] | 34.8 [32.7,36.9] | -1.1 [-2.7,+0.4] |
| Strongly agree | 18.3 [16.4,20.3] | 16.8 [15.0,18.6] | -1.5 [-3.7,+0.6] |

* Statistically significant differences (p<0.05) between responses at baseline and at follow-up.

^a^ Adjusted for LG and facility

^b^ “Yes” responses reported.

**Table S4: Comparison of awareness and knowledge between baseline and follow-up for customers from facilities that implemented the ‘limit sugary drinks’ nudge between baseline (2018, n = 871) and follow-up (2019, n =954) surveys**

| **Survey question and possible answers** | **Mean [95% CI]** | | **Percentage point difference in response between baseline and follow-up [95% CI]** |
| --- | --- | --- | --- |
|  | **Baseline (n = 871)** | **Follow-up (n = 954)** |  |
| **Awareness of change: Have you noticed changes to ^a^:** |  |  |  |
| The food and drink offered at the facility | 20.5 [17.7,23.3] | 23.4 [20.6,26.3] | +2.9 [-1.5,+7.3] |
| The variety of sugary drinks | 3.6 [2.4,4.8] | 7.8 [5.5,10.1] | +4.2 [+1.4,+7.0] * |
| The availability of sugary drinks | 2.7 [1.7,3.7] | 10.9 [8.1,13.9] | +8.2 [+5.0, +11.5] * |
| The fridge display | 8.9 [7.0,10.7] | 16.8 [13.9,19.7] | +7.9 [+4.2,+11.7] * |
| The price of drinks | 3.3 [2.0,5.1] | 5.7 [3.6,7.8] | +2.4 [-0.3,+5.2] |
| The variety of water | 1.8 [0.9,2.7] | 5.6 [3.7,7.6] | +3.8 [+1.5,+6.1] * |
| The availability of water | 1.7 [0.8,2.5] | 7.1 [4.3,9.8] | +5.4 [+2.3,+8.4] * |
| The advertising of water | 0.8 [0.2,1.3] | 5.2 [1.9,8.6] | +4.4 [+0.8,+8.1] * |
| **Knowledge related questions ^a^:** |  |  |  |
| *Is the following a healthy drink for everyday* |  |  |  |
| Water | 98.0 [96.8,99.1] | 97.9 [96.9,99.0] | -0.1 [-1.6,+1.6] |
| Juice | 15.0 [12.3,17.8] | 13.8 [11.5,16.1] | -1.2 [-5.2,+2.7] |
| Sugary drinks | 2.2 [0.9,3.5] | 1.6 [0.7,2.5] | -0.6 [-2.3,+1.0] |
| Diet sugary drinks | 2.2[1.2,3.2] | 5.8 [3.8,7.8] | +3.6 [1.2,+6.0] * |
| *Drinking sugary drinks often will* |  |  |  |
| Lead to weight gain | 90.8 [88.6,93.0] | 93.8 [92.2,95.5] | +3.0 [0.0,+6.1] |
| Increase your risk of heart disease | 74.3 [71.1,77.5] | 79.3 [76.4,82.1] | +5.0 [0.3,+9.7] * |
| Have no impact on your health | 9.6 [7.4,11.9] | 8.7 [6.7,10.8] | -0.9 [-4.3,+2.5] |
| Increase your risk of diabetes | 85.3 [82.8,87.8] | 84.9 [82.4,87.5] | -0.4 [-4.3,+3.5] |
| Help you live a healthier life | 3.2 [1.8,4.6] | 3.9 [2.4,5.3] | +0.7 [-1.4,+2.8] |
| Increase your risk of dental decay | 86.2[83.7,88.8] | 86.0 [83.5,88.4] | -0.2 [-4.2,+3.6] |
| **Water consumption ^a^** |  |  |  |
| Drank water while at the facility on day of survey | 78.6 [75.5,81.7] | 81.4 [78.9,84.0] | +2.8 [-1.6,+7.2] |
| **Agreement with:** |  |  |  |
| *Sport and recreation facilities have a responsibility to promote healthy eating* | | |  |
| Strongly disagree | 5.4 [4.3,6.5] | 6.6 [5.3,8.0] | +1.2 [+0.0,+2.4] |
| Disagree | 3.8 [2.9,4.7] | 4.5 [3.5,5.5] | +0.7 [+0.0,+1.4] |
| Neither disagree no agree | 12.2 [10.5,13.9] | 14.0 [12.2,15.9] | +1.8 [+0.0,+3.7] |
| Agree | 36.1 [33.7,38.4] | 37.3 [35.0,39.6.0] | +1.2 [+0.0,+2.5] |
| Strongly agree | 42.5 [39.1,46.0] | 37.5 [34.4,40.7] | -5.0 [-9.8,-0.1] |
| *Removing sugary drinks from sport and recreation facilities will lead to reduced consumption in the community* | | |  |
| Strongly disagree | 8.2 [6.8,9.7] | 9.8 [8.1,11.4] | +1.6 [-0.1,+3.2] |
| Disagree | 14.0 [12.2,15.9] | 15.9 [13.9,17.8] | +1.9 [-1.7,+3.8] |
| Neither disagree no agree | 22.7 [20.6,24.7] | 23.9 [21.8,25.9] | +1.2 [-0.1,+2.6] |
| Agree | 36.3 [33.9,38.7] | 34.4 [32.0,36.8] | -1.9 [-3.9,+0.2] |
| Strongly agree | 18.8 [16.4,21.3] | 16.2 [14.1,18.2] | -2.6 [-5.5,+0.2] |

Note, surveys where the facilities were unknown were excluded from the analysis (n = 309)

* Statistically significant differences (p<0.05) between responses at baseline and at follow-up.

^a^ “Yes” responses reported.

### **Table S5: Characteristics of staff survey respondents at baseline (2018, n = 162) and follow-up (2019, n = 183)**

| **Characteristic** | **(n (%))** | |
| --- | --- | --- |
|  | **Baseline** | **Follow-up** |
| **Job description** | n = 186 | n = 200 |
| *Employed by council* |  |  |
| Senior management | 14 (7.5) | 19 (9.5) |
| Health promotion | 26 (14.0) | 30 (15.0) |
| Sport and recreation | 47 (25.3) | 36 (18.0) |
| *Employed by sport and recreation facility* |  |  |
| Management | 20 (10.8) | 32 (16.0) |
| Customer service | 27 (14.5) | 40 (20.0) |
| Volunteer (management) | 8 (4.3) | 6 (3.0) |
| Volunteer (customer service) | 2 (1.1) | 5 (2.5) |
| Other (e.g. lifeguard) | 42 (22.6) | 32 (16.0) |
| **Time in role** | n = 186 | n = 202 |
| Less than 6 months | 29 (15.6) | 28 (13.9) |
| 6 months to 1 year | 38 (20.4) | 35 (17.3) |
| More than 1 year | 21 (11.3) | 32 (15.8) |
| 2 years or more | 98 (52.7) | 107 (53.0) |
| **Role in making healthy retail changes** | n = 165 | n = 190 |
| Yes | 59 (35.7) * | 102 (53.8) * |
| **Role related to:** |  |  |
| *Implementing the change* | n = 117 | n = 190 |
| Yes | 33 (28.2) | 57 (30.0) |
| No | 84 (71.8) | 133 (70.0) |
| *Policy development* | n = 116 | n = 190 |
| Yes | 24 (20.7) | 36 (18.9) |
| No | 92 (79.3) | 154 (81.0) |
| *Customer discussion* | n = 114 | n = 190 |
| Yes | 26 (22.8) | 36 (18.9) |
| No | 88 (77.2) | 154 (81.0) |
| **Purchase frequency from facility café** | n = 186 | n = 202 |
| Never | 61 (32.8) | 45 (22.3) |
| Less than once per week | 27 (14.5) | 39 (19.3) |
| 1-4 times per week | 95 (51.1) | 115 (56.9) |
| ≥5 times per week | 3 (1.6) | 3 (1.5) |
| **SEIFA quintiles ^a^** | n = 220 | n = 235 |
| 1 (most disadvantaged) | 89 (40.5) * | 70 (29.8) * |
| 2 | 33 (15.5) * | 67 (28.5) * |
| 3 | 77 (35.0) * | 68 (28.9) * |
| 4 | 21 (9.5) * | 30 (12.8) * |
| 5 (least disadvantaged) | 0 (0.0) * | 0 (0.0) * |

*Statistically significant differences (p<0.05) between responses at baseline and at follow-up.

^a^ LGs are ranked from most disadvantaged (1) to least disadvantaged (10) using the decile rank within Australia. Australian Bureau of Statistics. Census of Population and Housing: Socio-Economic Indexes for Areas (SEIFA) 2016. [Internet]. Canberra, Australia. 2021. [cited 2021 July]. Available from: https://www.abs.gov.au/ausstats/abs@.nsf/mf/2033.0.55.001

### **Table S6: Comparison of staff knowledge regarding organisational intent to implement healthy changes and awareness of polices related to the intervention at baseline in Water in Sport intervention councils (2018, n = 162) and follow-up (2019, n = 183)**

| **Survey question and possible answers** | **Baseline (n = 162)** | **Follow-up (n = 183)** | **p-value, comparison between baseline and follow-up** |
| --- | --- | --- | --- |
|  | **n (%)** | |  |
| **Organisational intent** |  |  |  |
| *Promoting healthy eating is a:* |  |  |  |
| Low priority | 22 (13.6) | 19 (10.4) | 0.242 |
| Medium priority | 72 (44.4) | 71 (38.8) |  |
| High priority | 68 (42.0) | 71 (38.8) |  |
| *Compared to one year ago, your organisational priority to promote healthy eating:* | | |  |
| Decreased | 10 (6.2) | 3 (1.6) | 0.011 |
| Stayed the same | 45 (27.8) | 34 (18.6) |  |
| Increased | 87 (53.7) | 126 (68.9) |  |
| Unsure | 20 (12.4) | 20 (10.9) |  |
| *Which best describes your organisation’s intent on making healthy changes:* | | |  |
| Not thought about it | 12 (7.5) | 5 (2.8) | <0.001 |
| Thinking about it | 19 (11.9) | 13 (7.1) |  |
| Preparing for change | 51 (31.9) | 11 (6.0) |  |
| Made changes within the past 6 months | 54 (33.8) | 63 (34.6) |  |
| Made changes within the past 6 months that are still in place | 22 (13.8) | 86 (47.3) |  |
| Made changes within the past 6 months that are no longer fully in place | 2 (1.3) | 4 (2.2) |  |
| **Agreement with:** |  |  |  |
| *Sport and recreation facilities have a responsibility to promote healthy eating* | | | |
| Strongly disagree | 5 (3.1) | 7 (3.8) | 0.857 |
| Disagree | 6 (3.7) | 7 (3.8) |  |
| Neither disagree no agree | 17 (10.5) | 26 (14.2) |  |
| Agree | 61 (37.7) | 66 (36.1) |  |
| Strongly agree | 73 (45.1) | 77 (42.1) |  |
| *Removing sugary drinks from sport and recreation facilities will lead to reduced consumption in the community* | | | |
| Strongly disagree | 6 (3.7) | 10 (5.5) | 0.902 |
| Disagree | 21 (13.0) | 22 (12.0) |  |
| Neither disagree no agree | 35 (21.6) | 43 (23.5) |  |
| Agree | 73 (45.1) | 76 (41.5) |  |
| Strongly agree | 27 (16.7) | 32 (17.5) |  |
| **Awareness of policy change related to: ^a^** |  |  |  |
| No sugary drinks sold | 1 (0.6) | 13 (7.1) | 0.002 |
| Sugary drinks must be hidden from view | 12 (7.5) | 20 (10.9) | 0.268 |
| Reducing the amount of sugary drinks for sale | 50 (31.1) | 108 (59.0) | <0.001 |
| No advertising of sugary drinks | 16.8 (27) | 33.9 (62) | <0.001 |
| Decreasing the price of water | 12 (7.5) | 22 (12.0) | 0.157 |
| Increasing the availability of water | 47 (29.2) | 94 (51.4) | <0.001 |
| **Support for council making new/additional healthy changes** | 151 (93.2) | 171 (93.4) | 0.931 |

^a^ “Yes” responses reported.

**Table S7: Comparison of staff knowledge regarding organisational intent to healthy changes and awareness of polices related to the healthy change intervention at baseline and follow-up for staff from facilities where a ‘limit sugary drinks’ nudge was implemented between baseline and follow-up surveys**

| **Survey question and possible answers** | **Mean [95% CI]** | | **Difference in response between baseline and follow-up (%)** |
| --- | --- | --- | --- |
|  | **Baseline (n = 83)** | **Follow-up (n = 97)** |  |
| **Organisational intent:** |  |  |  |
| *Promoting healthy eating is a:* |  |  |  |
| Low priority | 17.6 [10.5, 24.6] | 11.7 [6.6, 16.8] | -5.9 [-12.9, +1.1] |
| Medium priority | 43.6 [35.7, 51.6] | 38.0 [30.3, 45.4] | -5.6 [-12.6, +1.0] |
| High priority | 38.7 [28.8, 48.7] | 50.5 [41.1, 60.0] | +11.8 [-1.5, +25.0 |
| *Compared to one year ago, has the above:* |  |  |  |
| Decreased | 5.8 [2.0, 9.5] | 3.9 [1.2, 6.5] | -1.9 [-4.2, +0.3] * |
| Stayed the same | 24.8 [17.8, 31.9] | 19.4 [13.3, 25.4] | -5.5 [-11.1, +0.1] * |
| Increased | 53.2 [43.0, 63.4] | 67.1 [58.0, 76.0] | +13.9 [+0.4, +2.74] * |
| Unsure | 16.2 [9.4, 22.7] | 9.7 [5.0, 14.4] | -6.5 [-13.0, 0.0] * |
| *Which best describes your organisation’s intent on making healthy changes:* | | |  |
| Not thought about it | 11.2 [5.2, 17.1] | 3.5 [1.2, 5.7] | -7.7 [-12.6, -2.8] * |
| Thinking about it | 13.5 [7.3, 19.7] | 5.1 [2.2,8.0] | -8.4 [-13.3, +3.5] * |
| Preparing for change | 17.8 [11.2, 24.6] | 8.9 [4.9, 12.9] | -8.9 [-14.0, +3.9] * |
| Made changes within the past 6 months | 38.4 [30.8, 46.0] | 37.4 [30.1, 44.8] | -0.9 [-6.2, +4.2] * |
| Made changes more than past 6 months that are still in place | 18.3 [11.3,25.4] | 42.7 [33.6, 51.9] | +24.4 [+14.0, +34.8] * |
| Made changes more than past 6 months that are no longer fully in place | 0.7 [-0.1, 1.5] | 2.4 [-0.3, 5.0] | +1.7 [-2.8, +3.7] * |
| **Awareness of policy change related to:** | | |  |
| No sugary drinks sold | No affirmative responses | 6.9 [2.0, 11.9] |  |
| Sugary drinks must be hidden from view | 6.8 [1.6, 12.3] | 5.9 [1.3, 10.5] | -1.1 [-8.1, +6.0] |
| Reducing the amount of sugary drinks for sale | 34.9 [24.8, 45.0] | 58.2 [48.9,67.5] | +23.5 [+9.6, +37.4] * |
| No advertising of sugary drinks | 24.4 [15.3, 33.5] | 35.6 [26.3, 45.0] | +11.2 [-1.8, +24.2] |
| Decreasing the price of water | 10.5 [3.9, 17.0] | 8.9 [3.4, 14.5] | -1.6 [-10.8, +7.0] |
| Increasing the availability of water | 31.4 [21.6, 41.2] | 45.5 [36.0, 55.3] | +14.1 [+0.3, +28.0] * |
| **Agreement with:** |  |  |  |
| *Sport and recreation facilities have a responsibility to promote healthy eating* | | |  |
| Strongly disagree | 4.2 [1.1, 7.3] | 4.6 [1.3, 8.0] | +0.4 [-1.9, +2.7] |
| Disagree | 5.9 [2.2, 9.5] | 6.3 [2.2, 9.5] | +0.4 [-2.3, +3.3] |
| Neither disagree no agree | 15.0 [9.2, 20.9] | 16.0 [10.0, 22.0] | +1.0 [-4.4, +6.2] |
| Agree | 44.2 [37.1, 51.6] | 44.5 [37.2, 51.8] | +0.3 [-0.8, +1.1] |
| Strongly agree | 30.5 [21.4, 39.5] | 28.5 [20.1, 36.9] | -2.0 [-1.3, +9.3] |
| *Removing sugary drinks from sport and recreation facilities will lead to reduced consumption in the community* | | |  |
| Strongly disagree | 6.7 [2.5, 10.8] | 5.6 [2.1, 9.1] | -1.1 [-4.1, +2.1] |
| Disagree | 18.4 [11.6, 25.2] | 16.2 [10.2, 22.2] | -2.2 [-8.7, +4.3] |
| Neither disagree no agree | 30.1 [23.1, 35.7] | 28.9 [22.0, 35.7] | -1.2 [-4.9, +2.5] |
| Agree | 35.7 [27.4, 44.0] | 38.5 [30.4, 46.7] | +2.8 [5.7, +11.3] |
| Strongly agree | 9.2 [4.4, 13.9] | 10.7 [5.5, 15.9] | +1.5 [-3.2, +6.3] |
| **Support for council making new/additional healthy changes** | 88.0 [80.1, 95.0] | 94.8 [90.4, 99.0] | +96.8 [-1.4, +15.2] |

Note, surveys where the facilities were unknown were excluded from the analysis (n = 193), a further 54 were excluded depending on nudge implementation

* Statistically significant differences (p<0.05) between responses at baseline and at follow-up.
